# The preferred surgical choice for intermediate-risk papillary thyroid cancer: total thyroidectomy or lobectomy? A systematic review and meta-analysis

**DOI:** 10.1097/JS9.0000000000001556

**Published:** 2024-05-13

**Authors:** Mingyu Cao, Tiexin Yu, Xingyu Miao, Zhijing Wu, Wenlong Wang

**Affiliations:** a Department of General Surgery, Xiangya Hospital, Central South University; b Clinical Research Center for Breast Cancer Control and Prevention in Hunan Province, Changsha; c National Clinical Research Center for Geriatric Disorders, Xiangya Hospital of Central South University, Changsha, Hunan, People’s Republic of China

**Keywords:** intermediate-risk, lobectomy, papillary thyroid carcinoma, total thyroidectomy

## Abstract

**Background:**

The optimal surgical approach for intermediate-risk papillary thyroid carcinoma (IR-PTC) (according to ATA definition), whether total thyroidectomy (TT) or lobectomy (LT), has remained a contentious clinical gray area for several decades. This systematic review and meta-analysis aim to provide robust evidence and address this clinical dilemma comprehensively.

**Materials and methods:**

A comprehensive literature search was conducted in Pubmed, Embase, Web of Science, and the Cochrane Library from 1st January 2009 to 29th December 2023 to evaluate the impact of different surgical options (TT or LT) on patients with IR-PTC. The primary outcomes included survival, recurrence rates, and postoperative complications. *I* ^2^ and sensitivity analysis was used to explore the heterogeneity.

**Results:**

A total of 8 studies involving 2984 participants were included in this meta-analysis and systematic review. The results indicated that LT was a superior choice for mitigating complications compared to TT [risk ratio (RR), 0.32; 95% CI: 0.24–0.44, *P*<0.01], particularly for transient complications (RR, 0.24; 95% CI: 0.08–0.65, *P*<0.01), such as the transient parathyroid dysfunction (RR, 0.04; 95% CI: 0.01–0.15, *P*<0.01). However, TT did not increase the risk of recurrent laryngeal nerve palsy (RR, 0.78; 95% CI: 0.24–2.47, *P*=0.67), hemorrhage/seroma (RR, 0.77; 95% CI: 0.48–1.25, *P*=0.30), and permanent complications (RR, 0.18; 95% CI: 0.02–1.42, *P*=0.10). Besides, both LT and TT presented similar effect on survival outcomes (overall survival: RR, 1.00; 95% CI: 0.97–1.03, *P*=0.92, disease-specific survival: RR, 0.99; 95% CI: 0.97–1.02, *P*=0.69, recurrence-free survival: RR, 1.00; 95% CI: 0.96–1.05, *P*=0.86), recurrence (RR, 1.05; 95% CI: 0.76–1.46, *P*=0.76).

**Conclusion:**

The present meta-analysis revealed that TT did not yield improved outcomes in IR-PTC patients, but was associated with an increased incidence of temporary complications. In light of these findings, it may be advisable to consider LT as the optimal choice for IR-PTC patients.

## Introduction

Highlights

- Compared with lobectomy, total thyroidectomy does not improve overall survival, disease-specific survival, recurrence-free survival, and recurrence rates in patients with intermediate-risk papillary thyroid carcinoma (IR-PTC).
- In patients with IR-PTC, total thyroidectomy significantly increases the incidence of transient parathyroid disfunction but does not elevate the risk of permanent complications.
- This is the first meta-analysis demonstrates that lobectomy is the best surgical choice for patients with IR-PTC.

The incidence of papillary thyroid carcinoma (PTC) has significantly increased over the past few decades, with a nearly threefold rise since 1975^1^. Surgical intervention is the primary treatment for PTC, typically involving either lobectomy (LT) or total thyroidectomy (TT), depending on disease extent^2,3^. The 2015 American Thyroid Association (ATA) guidelines refined risk stratification for PTC patients into low-risk, intermediate-risk, and high-risk groups^4^. Currently, there is a preliminary consensus that LT is the preferred treatment for low-risk PTC, while TT is recommended for high-risk cases^5–7^. However, controversy surrounds the surgical management of intermediate-risk papillary thyroid carcinoma (IR-PTC), and an optimal approach has yet to be established.

Although the 2009 ATA guidelines initially recommended TT for PTC patients with tumor size larger than 1 cm^8^, the updated version in 2015 revised this recommendation to include both TT and LT as acceptable options^4^. Inconsistent recommendations have caused confusion among surgeons potentially impacting patient outcomes. Consequently, numerous studies have been conducted to determine the optimal surgical approach for IR-PTC; however, available results of the retrospective cohort studies are conflicting without a consensus^9–16^. Therefore, higher quality evidence is urgently needed to draw more persuasive conclusions.

Currently, there is a dearth of high-quality evidence focusing on the IR-PTC population. To our knowledge, no reviews have assessed the optimal surgical choice between LT and TT for patients with IR-PTC. Therefore, we conducted a comprehensive systematic review and meta-analysis to address this research gap. Our study aimed to investigate the differential effects of LT and TT on patients with IR-PTC, including subgroup analyses based on various outcomes such as survival, recurrence, and complications. We anticipate that our findings will provide precise surgical recommendations for patients with IR-PTC.

## Material and methods

### Protocol and literature search strategy

This meta-analysis and systematic review was reported according to the Preferred Reporting Items for Systematic Reviews and Meta-Analyses (PRISMA) recommendations (Supplemental Digital Content 1, http://links.lww.com/JS9/C502, 2, http://links.lww.com/JS9/C503) and in compliance with Assessing the methodological quality of systematic reviews (AMSTAR) criteria (Supplemental Digital Content 3, http://links.lww.com/JS9/C504)^17,18^. The protocol was registered on 22 January 2024, with PROSPERO (CRD42024501306) and also researchregistry.com (reviewregistry1821) prior to conducting this meta-analysis and systematic review. Pubmed, Embase, Web of Science, and the Cochrane Library were utilized to identify eligible clinical studies from 1st January 2009 to 29th December 2023. The focus was on surgical approaches for IR-PTC, using the following search terms: (‘thyroid carcinoma’ OR ‘thyroid cancer’ OR ‘thyroid neoplasm’ OR PTC OR TC) AND (‘intermediate*risk’ OR ‘risk’) AND (‘total thyroidectomy’ OR ‘complete thyroidectomy’ OR TT OR CT) AND (‘lobectomy’ OR ‘hemithyroidectomy’ OR LT or HT). Snowball sampling was employed to identify additional reviews or studies not found in these databases by screening references of included studies and consulting with experts.

### Study inclusion and exclusion criteria

Study inclusion criteria encompassed randomized controlled trials, observational studies, and clinical trials comparing TT and LT in IR-PTC patients. Eligible research included PTC patients diagnosed with intermediate-risk based on ATA risk stratification, along with detailed information regarding their surgical approach of either TT or LT. Excluded were articles lacking explicit risk stratification, unclear descriptions of surgical methods, incomplete baseline characteristics, and inadequate outcome records pertaining to survival rates, recurrence rates, or complications. Additionally excluded were letters to the editor, editorials, conference papers, as well as articles published in languages other than English. Published meta-analyses and systematic reviews related to the topic were only consulted for data if any omissions occurred. In cases where eligible studies had overlapping or redundant participant cohorts, we extracted and analyzed the most recent and largest population data unless there was a concern regarding study design quality. When encountering studies presenting both pre-Propensity Score Matching and post-Propensity Score Matching (PSM) data sets, priority was given to post-PSM data first. For studies without PSM analysis; their data was also considered for pooling during synthesis.

### Study selection and data extraction

The study selection and data extraction of eligible studies were independently conducted by three reviewers under the supervision of the field’s expert. The study selection process consisted of four stages. Firstly, duplications were removed. Secondly, titles and abstracts of potential eligible researches were assessed based on the inclusion/exclusion criteria. Thirdly, full texts underwent comprehensive screening. Lastly, data extraction and quality assessment were performed. Disagreements among authors were resolved through discussion. Study selection was carried out following the PRISMA protocol^19^. For each eligible study, the extracted data included: first author, year of publication, number of participants in each group (TT and LT), baseline characteristics of included patients, main outcome events (survival, recurrence, and complications), as well as items used for quality assessment.

### Quality assessment

The methodological quality assessment was independently conducted by three reviewers using the Newcastle–Ottawa Scale (NOS), a reliable tool for evaluating evidence. The eligible studies were classified as ‘low level’, ‘medium level’, or ‘high level’ based on three aspects of bias assessment. In case of any disagreement, a consensus was reached.

### Definitions

According to the 2015 ATA guideline, the IR-PTC was defined as patients with those exhibiting microscopic soft-tissue invasion, aggressive histology, lymph node metastases (clinical N1 or >5 pathologic N1 with all involved lymph nodes <3 cm in largest dimension) or uptake outside the thyroid bed on radioactive iodine (RAI) scans^4^. As for the complications, hypocalcemia and hypoparathyroidism are characterized by the cessation of exogenous calcium and vitamin D, levels of blood calcium or PTH lower than normal, or symptoms indicative of low calcium. Recurrent laryngeal nerve palsy usually refers to a neurological disorder of the larynx caused by abnormal movement of one or both vocal cords. The complications are temporary if recovered within 6 months, and permanent after 6 months^16,20,21^.

### Statistical analysis

The fixed effect model was primarily employed for estimation, and the *I* ^2^ statistic was also utilized to assess heterogeneity. If the *I* ^2^ value exceeded 50%, then a high heterogeneity is indicated, a random effect model would be used as an alternative to the fixed one so as to obtain more precise results^22–24^. Subgroup analyses were conducted to identify potential sources of heterogeneity and mitigate publication bias. Statistical analyses were performed using Review Manager (RevMan), version 5.4, with the risk ratio (RR) set as the effect measure for outcomes. A significance level of 0.05 was applied to all analyses. Funnel plots were employed when including more than 10 studies to evaluate publication bias.

## Results

### Study selection, characteristics, and quality assessment

**Figure 1:**
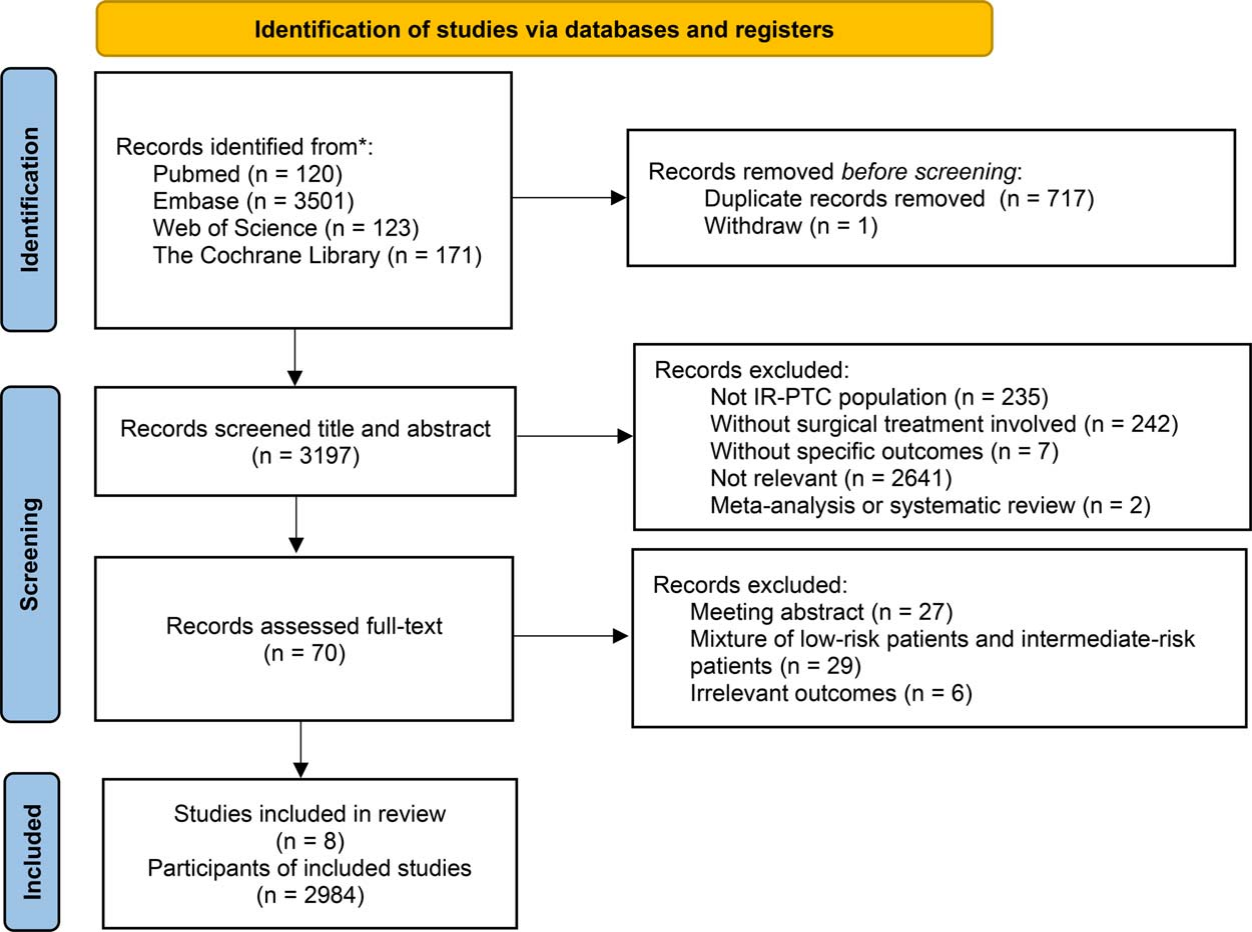
PRISMA flowchart.

The database searches yielded a total of 3915 records, which were subsequently screened for duplicates and withdrawn records. Following this, the titles and abstracts were thoroughly reviewed. Ultimately, a comprehensive evaluation was conducted on 70 studies based on full-text analysis, resulting in the inclusion of 8 retrospective cohort studies with a combined patient population of 2984 individuals for this systematic review and meta-analysis (Fig. 1). The baseline characteristics of these eight retrospective cohort studies are summarized in Table 1, including country, article type, sample size, sex, mean age, detailed surgical treatment criteria of each group, follow-up duration, outcomes of interest, and final conclusion. Among them, a total of 1771 patients with IR-PTC underwent TT, while 1213 patients with IR-PTC underwent LT, and 70.38% patients are female. Furthermore, the most frequently reported outcomes were survival^9,12,15,16,20,25,26^ and recurrence^9,11,15,16,20,25,26^, with postoperative complications also being of interest in five studies^11,16,20,25,26^. Seven of these studies provided data from Asia^9,12,15,16,20,25,26^, while only one study was conducted in Italy^11^. The mean follow-up duration was 10.38±7.30 years, which indicated a considerably satisfactory follow-up time and lent credibility to the results.

**Table 1:** Baseline characteristics of included studies.

Title	First author (Years)	Country	Article type	Sample size (TT/LT)	Case (female/male)	Mean Age (years)	Participants	Intervention	Comparator	follow-up (years)	Outcomes	Results (favor which)	OS (TT/LT)	DSS (TT/LT)	RFS (TT/LT)	Recurrence (TT/LT)	Complication (TT/LT)
Total vs hemithyroidectomy for intermediate-risk papillary thyroid cancer: a 23 years retrospective study in a tertiary center	Tsui, K.P.(2019)	Hong Kong, China	Retrospective cohort study	137 (92/45)	137 (66/71)	53	Patients with follicular variant, oxyphilic cell variant, a diffuse sclerosing variant or tall cell variant were excluded. Patients who underwent subtotal thyroidectomy or presented with distant metastases were also excluded from analysis. A total of 137 patients were identified to be of intermediate-risk. The median follow-up time was 54 months	When a tumor was restricted to one lobe and there was no evidence of high-risk features, which included age >45 years, cervical lymph node metastases and gross extrathyroidal extension, hemithyroidectomy was performed. Patients with multiple tumors in one lobe were also treated by hemithyroidectomy as long as all tumors were removed	Total thyroidectomy was performed for patients who presented with tumors in both lobes or had high-risk features	22.8	1. DSS 2. RFS 3. recurrence 4. complication	1. DSS: no favor 2. RFS: favor LT 3. Recurrence (5 years): favor LT 4. complication rate: favor LT	NA	NA	130(86/44)	7(7/0)	35(32/3)
Long-term prognosis of unilateral and multifocal papillary thyroid microcarcinoma after unilateral lobectomy versus total thyroidectomy	Jeon, Y. W.(2019)^20^	Korea	Retrospective cohort study	255 (128/127)	255 (226/29)	49	All patients had a diagnosis of unilateral multifocal PTMC. Unilateral multifocality was defined as two or more PTMC lesions present within only the ipsilateral lobe, which was confirmed by a head and neck pathologist at St. Vincent’s Hospital, The Catholic University, Korea. Patients were ineligible if they met the following criteria: tumor size [1 cm, bilateral multifocality, non-PTC carcinoma (follicular, medullary, anaplastic), high-risk histological subtypes (tall cell, columnar, and solid variants of PTC), gross ETE, CLN (central and lateral) metastasis according to the final pathological reports, postoperative radioactive iodine (RAI) therapy, and the presence of dis- tant metastasis	Lobectomy	Total thyroidectomy	17.4	1. RFS 2. recurrence 3. complication	RFS: no favor	NA	NA	250 (127/123)	5 (1/4)	25 (23/2)
Efficacy of hemithyroidectomy in papillary thyroid carcinoma with minimal extrathyroidal extension	Ji, Y. B.(2019)^26^	Korea	Retrospective cohort study	255 (173/82)	255 (201/54)	49	To include only unilateral cN0 PTC patients of less than 4 cm with minimal ETE, we excluded 1484 of the 1826 PTC patients who had tumors ≥4 cm, preoperative clinical lymph node metastasis, bilateral carcinoma, no ETE, maximal ETE, distant metastasis, or who underwent revision/completion thyroidectomy or concurrent lateral selective neck dissection. we also excluded those with tumors >2 cm or tumors with gross invasion of the sternothyroid muscle because only one patient with tumor >2 cm underwent hemithyroidectomy, and all the patients with gross invasion of the sternothyroid muscle had received total thyroidectomies. we included 255 PTC patients with unilateral cN0 tumors ≤2 cm and minimal ETE	Lobectomy	Postoperative radioactive iodine (RAI) ablation was performed in all patients with maximal ETE regardless of tumor size, tumor size larger than 4 cm, or distant metastases, and also in some selected patients with minimal ETE or cervical lymph node metastasis, and higher risk histologic features among those who underwent total thyroidectomy	5.5	1. complication 2. recurrence 3. RFS	1. transient complication: favor LT 2. recurrence: no favor 3. RFS: no favor	NA	NA	129 (64/65)	3 (2/1)	86 (61/25)
Total thyroidectomy versus lobectomy for intermediate-risk papillary thyroid carcinoma: A single-institution matched-pair analysis	Liu, J.(2019)^15^	China	Retrospective cohort study	682 (341/341)	682 (404/278)	NA	Patients aged 18 years or older diagnosed with PTC from January 1996 to December 2008 were first included, Patients with multiple cancer diagnoses were excluded to ensure that outcomes were not confounded by other cancer diagnoses and/or treatments; aggressive variants such as tall cell, columnar, and poorly differentiated PTC were also excluded. including microscopic invasion of the tumor into perithyroidal soft tissues (T1-2NxM0); clinical N1 stage or >5 pathologic N1lymph nodes with all involved lymph nodes <3 cm in the largest dimension (T1-2N1M0) and vascular invasion. BRAF V600E status and postoperative radioactive iodine (RAI) scans were not evaluated due to insufficient information in most cases. Patients with high-risk factors such as presence of distant metastasis or incomplete resection were excluded	Lobectomy	Cases involving near-total thyroidectomy, subtotal thyroidectomy, partial thyroidectomy were also excluded	16	1. Recurrence 2. RFS 3. DSS	1. Local recurrence: favor TT 2. regional and distant recurrence: no favor 3. RFS: no favor 4. DSS: no favor	668 (335/333) cause of death: 14 died from PTC	668 (335/333)	538 (274/264)	72 (35/37)	NA
Outcomes of patients with an intermediate-risk group according to the Japanese Risk Classification of Papillary Thyroid Carcinoma	Horiuchi, K.(2023)^12^	Japan	Retrospective cohort study	297 (169/128)	297 (221/76)	49	The patients classified as neither low (tumor diameter = <2 cm and negative for lymph node metastasis) nor high-risk (tumor diameter >5 cm, or palpable lymph node metastasis larger than 3 cm in diameter, or extra-thyroid extension beyond the membrane to the trachea/esophagus) groups belong to the intermediate-risk group	LT+lymph node dissection	TT+lymph node dissection	8	1.DFS	DFS: no favor	8 (7/1) cause of death: 289 died from PTC	8 (7/1)	NA	NA	NA
Comparison of lobectomy vs total thyroidectomy for intermediate-risk papillary thyroid carcinoma with lymph node metastasis	Xu S.(2023)^9^	China	Retrospective cohort study	530 (265/265)	530 (377/153)	37	The inclusion criteria were patients with PTC and clinical ipsilateral positive lymph nodes identified on the lateral neck (cN1b) and sufficient follow-up data (more than 24 months). Patients with evidence of a contralateral lesion (primary or central lymph node), primary tumor size greater than 4 cm or gross extrathyroidal extension (gETE; stages T3-T4), metastatic lymph node size greater than 3 cm, inadequate lymph node yield (total harvested lymph nodes <20), distant metastasis (stage M1), second primary malignancies, or incomplete resection were excluded. Patients with aggressive histologic variants were also excluded. Patients who underwent lobectomy and TT were categorized into study and control groups, respectively	The lobectomy group included patients who underwent lobectomy with or without isthmectomy, and patients who underwent lobectomy with nodule enucleation were also included in the lobectomy group	The TT group included patients who underwent total, subtotal, or near-total thyroid resection	5	1. Recurrence 2. RFS	RFS: no favor	NA	NA	261 (126/135)	38 (17/21)	NA
Unilateral TNM t1 and t2 papillary thyroid carcinoma with lateral cervical lymph node metastasis: Total thyroidectomy or lobectomy?	Wang, Z. (2020)^25^	China	Retrospective cohort study	264 (104/160)	264 (191/73）	36	Unilateral TNM T1 and T2 PTC patients with lateral lymph node metastasis The exclusion criteria for this study were: (1) cancer foci in both thyroid lobe; (2) Tumor maximum diameter >4 cm; (3) definite extrathyroidal invasion (ETE); (4) metastatic lymph node diameter >3 cm; (5) Aggressive variants such as tall cell, columnar, and poorly differentiated PTC; (6) Not initial surgery; (7) Partial surgical resection and subtotal resection and other irregular surgical methods and patients lack of information	The surgical procedure was LT, and unilateral cervical lymph node dissection	The surgical procedure was TT, and unilateral cervical lymph node dissection	5	1. Recurrence 2. RFS 3. Complication	1. RFS: no favor 2. Complication: no favor	262 (103/159) cause of death: one person(TT group) died from other disease, one person(LT group) died from PTC	263 (104/159)	256 (100/156)	7 (3/4)	8 (5/3)
Hemithyroidectomy versus total thyroidectomy in the intermediate-risk differentiated thyroid cancer: the Italian Societies of Endocrine Surgeons and Surgical Oncology Multicentric Study	C. Dobrinja(2021)^11^	Italy	Retrospective cohort study	564 (499/65)	564 (414/150)	48	Eligible patients had to be older than 18 years, with ultrasonography (US) tumor diameter between 1 and 4 cm, proven intermediate‐risk DTC, preoperative cytological diagnoses of TIR3A or TIR3B. Exclusion criteria were previous thyroid surgery, familiar history of thyroid cancer, previous neck or upper mediastinum radiation, US and/or biochemical evidence of thyroiditis, bilateral multinodular goiter, and bilateral cancer. Patients with preoperative evidence of lymph node disease, those with extrathyroidal extension at clinical and/or ultrasonography (US), and tumors larger than 4 cm were not included in the analysis	Hemithyroidectomy removes one of the thyroid lobes, leaving the other intact	Total thyroidectomy is the surgical removal of the whole thyroid gland	3.3	1.Recurrence 2. Complication 3.reintervention	1. Recurrence: no favor 2. Complication: favor LT 3. re-intervention: favor TT	NA	NA	NA	3 (3/0)	94 (89/5)

DFS, disease free survival; DSS, disease-specific survival; LT, lobectomy; OS, overall survival; RFS, recurrence-free survival; TT, total thyroidectomy.

**Figure 2:**
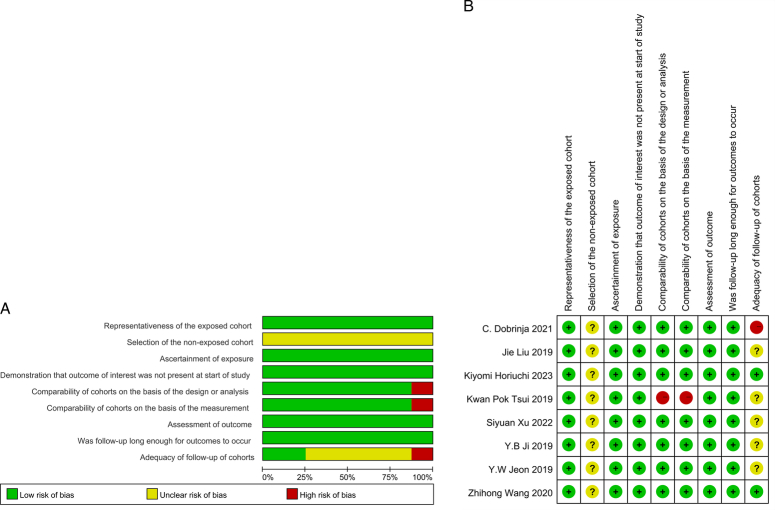
A. Risk of bias graph. B. Risk of bias summary.

Table 2 presented the quality assessment using the NOS scale for the eight eligible studies; seven were ranked as ‘high’ quality (score≥7), while one was ranked as ‘medium’ quality (6≥score≥4) (Fig. 2).

**Table 2:** Quality assessment.

			Quality assessment-NOS										
			Selection				Comparability		Outcome				
Title	First author (Year)	DOI	Representativeness of the exposed cohort	Selection of the nonexposed cohort	Ascertainment of exposure	Demonstration that outcome of interest was not present at start of study	Comparability of cohorts on the basis of the design or analysis	Comparability of cohorts on the basis of the measurement	Assessment of outcome	Was follow-up long enough for outcomes to occur	Adequacy of follow-up of cohorts	Total score	Rank
Total vs hemithyroidectomy for intermediate-risk papillary thyroid cancer: A 23 year retrospective study in a tertiary center	Calvin, T. K. P. (2018)	10.1016/j.amjoto.2019.04.001	1	0.5	1	1	0	0	1	1	0.5	6	Mid
Long-term prognosis of unilateral and multifocal papillary thyroid microcarcinoma after unilateral lobectomy versus total thyroidectomy	Jeon, Y. W.(2019)^20^	10.1245/s10434-019-07482-w	1	0.5	1	1	1	1	1	1	0.5	8	High
Efficacy of hemithyroidectomy in papillary thyroid carcinoma with minimal extrathyroidal extension	Ji, Y. B.(2019)^26^	10.1007/s00405-019-05598-z	1	0.5	1	1	1	1	1	1	0.5	8	High
Total thyroidectomy versus lobectomy for intermediate-risk papillary thyroid carcinoma: A single-institution matched-pair analysis	Liu, J.(2019)^15^	10.1016/j.oraloncology.2019.01.010	1	0.5	1	1	1	1	1	1	0.5	8	High
Outcomes of patients with an intermediate-risk group according to the Japanese risk classification of papillary thyroid carcinoma	Horiuchi, K. (2023)^12^	10.1007/s00268-023-07073-7	1	0.5	1	1	1	1	1	1	1	8.5	High
Comparison of lobectomy vs total thyroidectomy for intermediate-risk papillary thyroid carcinoma with lymph node metastasis	Xu S.(2023)^9^	10.1001/jamasurg.2022.5781	1	0.5	1	1	1	1	1	1	0.5	8	High
Unilateral TNM t1 and t2 papillary thyroid carcinoma with lateral cervical lymph node metastasis: Total thyroidectomy or lobectomy?	Wang, Z. (2020)^25^	10.4158/EP-2020-0125	1	0.5	1	1	1	1	1	1	1	8.5	High
Hemithyroidectomy versus total thyroidectomy in the intermediate-risk differentiated thyroid cancer: the Italian Societies of Endocrine Surgeons and Surgical Oncology Multicentric Study	C. Dobrinja(2021)^11^	10.1007/s13304-021-01140-1	1	0.5	1	1	1	1	1	1	0	7.5	High

NOS, Newcastle–Ottawa Scale.

### Survival

**Figure 3:**
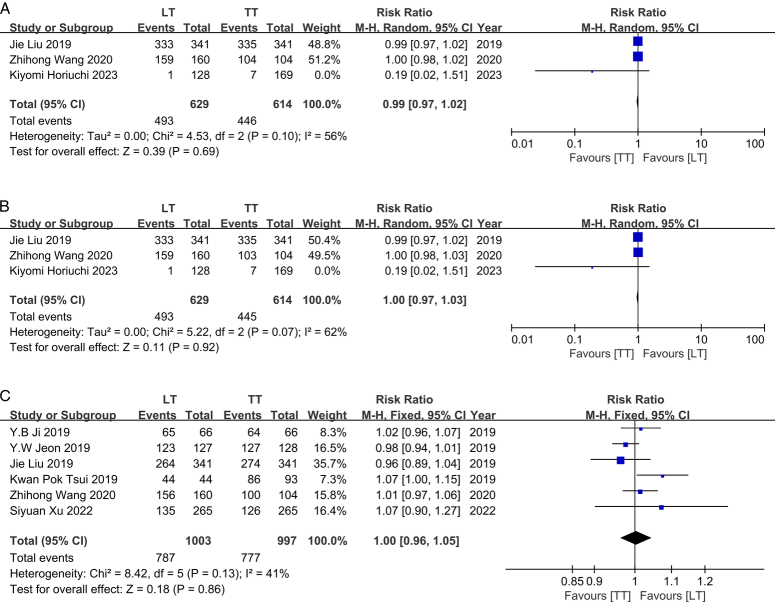
A. Forest plot showing effects of LT and TT on OS of IR-PTC patients. B. Forest plot showing effects of LT and TT on DSS of IR-PTC patients. C. Forest plot showing effects of LT and TT on RFS of IR-PTC patients.

The survival data of patients with IR-PTC were obtained from seven studies conducted between 2019 and 2023^9,12,15,16,20,25,26^. Among these records, a total of 1166 patients underwent TT, while 1131 patients underwent LT. Importantly, all seven individual studies reported no significant association between surgical approaches and survival outcomes. To enhance the persuasiveness of our findings, we further analyzed three subgroups based on overall survival (OS), disease-specific survival (DSS), and recurrence-free survival (RFS). No heterogeneity was observed in the RFS subgroup analyses (*I* ^2^=41%, *P*=0.13); therefore, a fixed-effects model was employed. However, substantial heterogeneity was detected among studies reporting OS and DSS data (OS: *I* ^2^=62%, *P*=0.07; DSS: *I* ^2^=56%, *P*=0.10), necessitating the use of a random-effects model instead. Our meta-analysis results across all three subgroups consistently indicated that there was no statistically significant difference in the survival rates of IR-PTC patients undergoing TT or LT procedures (OS: RR, 1.00; 95% CI: 0.97–1.03, *P*=0.92; DSS: RR, 0.99; 95% CI: 0.97–1.02, *P*=0.69; RFS: RR, 1.00; 95% CI: 0.96–1.05, *P*=0.86) (Fig. 3).

### Recurrence

**Figure 4:**
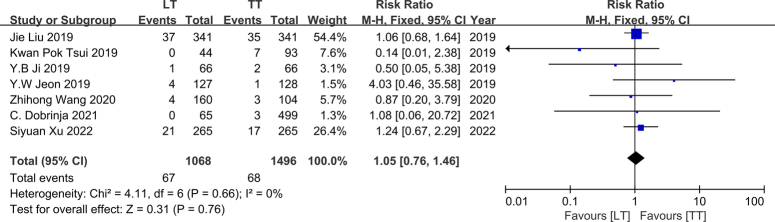
Forest plot showing effects of LT and TT on recurrence of IR-PTC patients.

Recurrence records were identified in seven studies conducted between 2019 and 2022^9,11,15,16,20,25,26^. A total of 1496 patients were included in the TT group, while the LT group comprised 1068 patients. All seven studies consistently reported a nonsignificant association between surgical method and recurrence rate. No heterogeneity was observed among these individual studies (*I* ^2^=0%, *P*=0.66), thus a fixed-effects model was employed. Consistent with these findings, our results also demonstrated that TT did not show a lower recurrence rate compared to LT (RR, 1.05; 95% CI: 0.76–1.46, *P*=0.76) (Fig. 4).

### Complications

**Figure 5:**
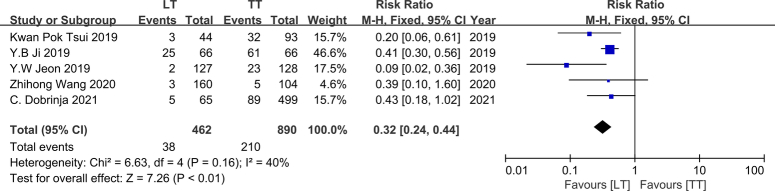
Forest plot showing effects of LT and TT on total complication occurrence of IR-PTC patients.

Postoperative complication data were extracted from five studies conducted between 2019 and 2021^11,16,20,25,26^. Among these citations, a total of 890 patients underwent TT, while 462 underwent LT. Notably, 60% (3/5) of the studies reported a significant association between LT and a lower incidence of complications^16,20,26^, whereas the remaining 40% (2/5) suggested similar postoperative complication rates for both TT and LT. These five studies exhibited no heterogeneity (*I* ^2^=40%, *P*=0.16); therefore, we employed a fixed-effects model in this analysis. Our findings indicate that LT was associated with fewer complications than TT (RR, 0.32; 95% CI: 0.24–0.44; *P*<0.01) (Fig. 5). To obtain more precise results, subgroup analyses were performed based on follow-up time (transient and permanent) and types of complications [recurrent laryngeal nerve palsy (RLNP), parathyroid dysfunction, and hemorrhage/seroma].

### Subgroup of complications – transient complications

**Figure 6:**
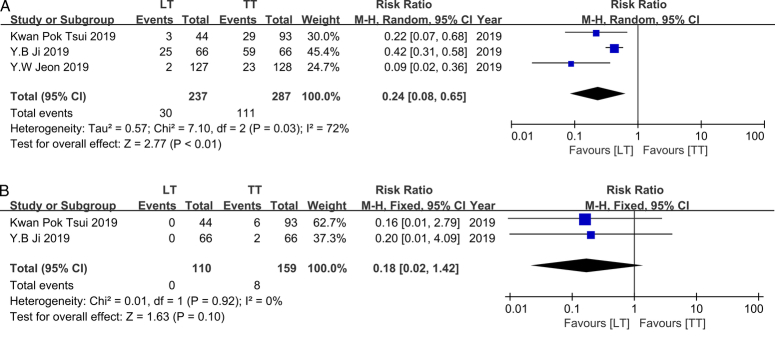
A. Forest plot showing effects of LT and TT on transient complication occurrence of IR-PTC patients. B. Forest plot showing effects of LT and TT on permanent complication.

In this meta-analysis, transient complications included transient RLNP, transient hypoparathyroidism/hypocalcemia, and hemorrhage/seroma. Three studies were evaluated in this section, all of which were published in 2019^16,20,26^. Across these references, the TT group comprised 287 patients while the LT group consisted of 237 patients. Consistently, all three studies indicated that LT outperformed TT in reducing the occurrence of transient complications. However, a substantial heterogeneity was observed (*I* ^2^=72%, *P*=0.03), necessitating the use of a random-effects model. Based on our findings, LT demonstrated a significantly lower incidence of transient complications compared to TT (RR, 0.24; 95% CI: 0.08–0.65; *P*<0.01), suggesting that LT may be a preferable option for mitigating such complications (Fig. 6A).

### Subgroup of complications – permanent complications

In this meta-analysis, permanent complications included permanent RLNP, permanent hypocalcemia, and permanent hypothyroidism. Two studies published in 2019^16,26^ were identified for harvesting records of permanent complications in this section. A total of 159 patients were classified into the TT group, while 110 patients were categorized into the LT group. Both studies demonstrated a nonsignificant association between surgical approach and occurrence of permanent complications, without any heterogeneity observed (*I* ^2^=0%, *P*=0.92). Therefore, a fixed-effects model was employed. Consequently, our meta-analysis did not identify any surgical preference to reduce the incidence of permanent complications (RR, 0.18; 95% CI: 0.02–1.42; *P*=0.10) (Fig. 6B).

### Subgroup of complications – RLNP

**Figure 7:**
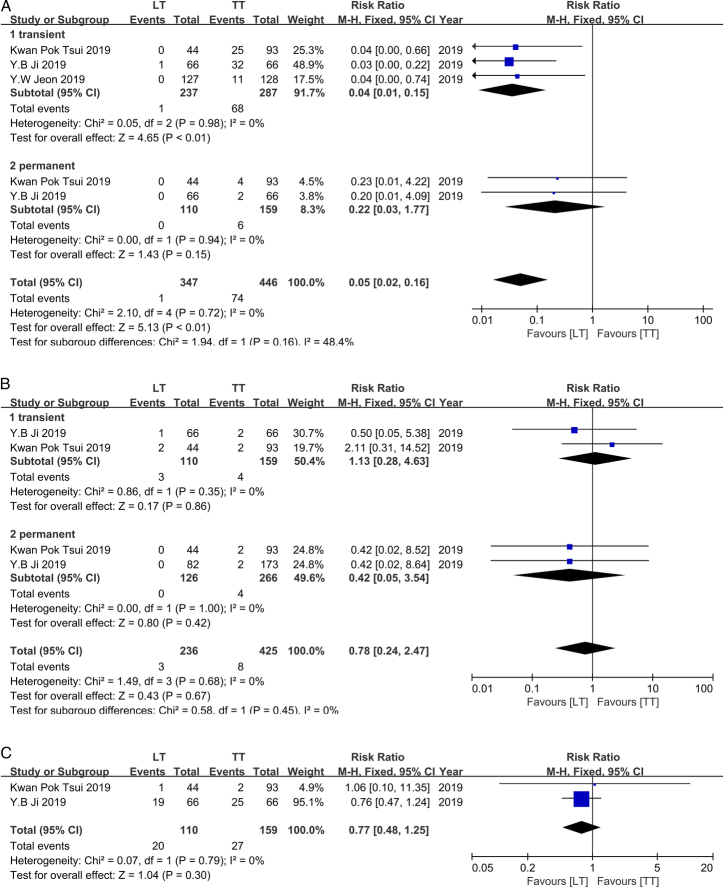
A. Forest plot showing effects of LT and TT on RLNP occurrence of IR-PTC Patients. B. Forest plot showing effects of LT and TT on parathyroid dysfunction. occurrence of IR-PTC patients. C. Forest plot showing effects of LT and TT on hemorrhage/seroma. occurrence of IR-PTC patients.

Only two studies in 2019 reported RLNP in IR-PTC^16,26^. These studies included a total of 425 RLNP cases after TT (transient/permanent: 159/266) and 236 RLNP cases after LT (transient/permanent: 110/126). Both individual studies indicated no surgical preference for avoiding RLNP, regardless of the transient or permanent scenario. Furthermore, there was no heterogeneity found between these studies (*I* ^2^=0%, *P*=0.68), thus a fixed-effect model was used. Our meta-analysis suggested that there was no statistically significant difference in the occurrence of RLNP between LT and TT, whether it was transient or permanent (Transient: RR, 1.13; 95% CI: 0.28–4.63, *P*=0.86; Permanent: RR, 0.42; 95% CI: 0.05–3.54, *P*=0.42; Overall effect: RR, 0.78; 95% CI: 0.24–2.47, *P*=0.67) (Fig. 7A).

### Subgroup of complications – parathyroid disfunction (hypoparathyroidism/hypocalcemia)

Parathyroid dysfunction records were reported in three studies conducted in 2019, involving a total of 446 patients who underwent TT (transient/permanent: 287/159) and 347 patients who underwent LT (transient/permanent: 237/110)^16,20,26^. Surprisingly, all the studies indicated that LT was associated with a reduced risk of temporary parathyroid dysfunction; however, this protective effect did not extend to permanent cases. Furthermore, there was no heterogeneity observed among the studies (*I* ^2^=0%, *P*=0.72), thus a fixed-effects model was employed. Consequently, our analysis supported these three findings that LT may decrease the risk of parathyroid dysfunction significantly (RR, 0.05; 95% CI: 0.02–0.16, *P*<0.01), particularly in temporary cases (RR, 0.04; 95% CI: 0.01–0.15, *P*<0.01). However, regarding permanent parathyroid dysfunction, there was no statistically significant difference between LT and TT (RR, 0.22; 95% CI: 0.03–1.77, *P*=0.15) (Fig. 7B).

### Subgroup of complications – hemorrhage/seroma

Only two studies published in 2019^16,26^ providing available records. In these two studies, a total of 159 patients underwent TT, while 110 patients underwent LT. Both studies reported no significant correlation between the surgical approach and the occurrence of hemorrhage/seroma. Furthermore, there was no heterogeneity observed among individual results (*I* ^2^=0%, *P*=0.79), leading to the adoption of a fixed-effects model for meta-analysis. Our meta-analysis also demonstrated that the choice between LT and TT did not associate with a reduced incidence of hemorrhage or seroma (RR, 0.77; 95% CI: 0.48–1.25, *P*=0.30) (Fig. 7C).

## Discussion

The surgical management of patients diagnosed with PTC, particularly those classified as intermediate-risk, remains a subject of ongoing debate regarding the choice between TT and LT. To our knowledge, this is the first meta-analysis demonstrating that TT does not yield improved outcomes in IR-PTC patients but is associated with an increased incidence of temporary complications. In light of these findings, it may be advisable to consider LT as a safer and effective alternative option for optimizing quality of life among IR-PTC patients.

The impact of surgical methods on the prognosis of patients, particularly in terms of long-term outcomes, remains a highly debated issue. Conducting RCT to evaluate the effect of different surgical approaches on IR-PTC prognosis is challenging due to the requirement for large sample sizes and extended follow-up periods^27^. IR-PTC exhibits a less indolent behavior compared to low-risk PTC, which can be effectively managed with LT and demonstrates low rates of recurrence or mortality^6^. Conversely, it is not as aggressive as high-risk PTC, typically necessitating TT but potentially leading to overtreatment-related complications that significantly impact patients’ quality of life^28^. It is noteworthy that TT has been the predominant surgical approach for IR-PTC for over a decade^29^. However, evolving comprehension of IR-PTC has resulted in revised surgical recommendations. According to the 2015 ATA guidelines, LT is now considered an acceptable alternative for IR-PTC management. LT may offer advantages over TT by reducing postoperative complications and enhancing patients’ quality of life, and the principle of ‘less is more’ is increasingly acknowledged in academic fields^4,30^.

Despite the theoretical advantages of a higher quality of life and avoidance of lifelong thyroid hormone replacement therapy associated with LT, the actual preference for complications remains unknown, while postoperative survival and recurrence status remain major concerns. This explains the current reliance on TT, as extensive radical surgery offers the greatest potential to prevent cancer-related death and recurrence and to perform adjuvant RAI treatment^31^. However, in light of rapid economic and social development, patients with IR-PTC are increasingly seeking maximum survival rates and postoperative quality of life while minimizing recurrence and complications. Surgeons also aim to avoid ‘overdiagnosis’ and ‘overtreatment’, which underscores the crucial value placed on striking a balance between LT and TT surgical approaches^32^. As early as 2019, evidence supported LT as the preferred choice for IR-PTC due to its superior performance in terms of 5-year recurrence-free survival compared to TT^16^. Nevertheless, a recent large-scale study has cast doubt on this claim by suggesting that there is no significant difference in recurrence-free survival between LT and TT for IR-PTC patients^9^. The discrepancies may arise from various factors, encompassing the inclusion and exclusion criteria, statistical methodologies, and physician and patient preferences. To address the aforementioned concerns, we conducted a comprehensive meta-analysis encompassing eight studies involving a total of 2984 participants and revealed no significant superiority of TT over LT in terms of OS, DSS, RFS, and recurrence rates. Surprisingly, Xu *et al*.^33^ demonstrated that the extent of surgery did not show any association with DSS in high-risk PTC patients. Following 1:1 case–control matching of 528 patients, no significant difference was observed between the LT and TT groups regarding the 10-year DSS rate or 10-year RFS rate. This finding enhances our confidence in the safety and efficacy of LT as a treatment modality for IR-PTC.

In the decision-making process of choosing between TT or LT for IR-PTC, it is crucial to consider postoperative complications, particularly permanent complications. Hypoparathyroidism and recurrent laryngeal nerve (RLN) injury are prevalent and significant complications associated with thyroidectomy^11^. The incidence of these complications inevitably increases in proportion to the extent of surgery^34,35^. A previous study revealed that the frequency and severity of complications after TT were significantly higher compared to those following LT, even when performed by highly experienced experts^1,36^. The present meta-analysis showed that TT significantly increased the incidence of transient parathyroid disfunction, but did not increase the risk of hemorrhage/seroma; unlike previous studies^37^, our results confirmed that TT did not increase the incidence of permanent complications. From the perspective of postsurgical complications, LT should be favored for IR-PTC.

However, some limitations that cannot be ignored when interpreting current study. First, the selected studies were all observational retrospective studies, which implies that the findings are constrained by the quality of available data and a substantial degree of heterogeneity. The gold standard methodology for addressing this question is to conduct a RCT. Patients with IR-PTC demonstrate excellent outcomes with prolonged survival times, conducting such a study would be impractical due to exorbitant costs and time constraints. Second, adjuvant therapies including thyroid hormone suppression therapy and RAI treatment, which were not consistently collected across different studies and have undergone changes over time. Third, the lack of available IR-PTC data comparing the effects of LT and TT represents a significant limitation. Most studies have combined patients with different risk levels, with few conducted exclusively among IR-PTC patients. However, since there is a lack of RCTs examining survival rates following TT and LT for IR-PTC, this current meta-analysis represents the most extensive study on outcomes and complications associated with these surgical procedures in patients with IR-PTC. Therefore, it serves as a crucial tool for determining the optimal surgical options within this specific patient population.

## Conclusion

In summary, our meta-analysis suggested that TT significantly increased the risk of temporary parathyroid dysfunction but failed to reduce recurrence rates and improve survival rates compared with LT for patients with IR-PTC, indicating that LT may be the optimal surgical methods for IR-PTC patients. Due to the limited research focusing on IR-PTC as a clinical ‘gray area’, the results must be interpreted with caution, further larger randomized clinical studies are necessary to validate our findings.

## Ethical approval

Not applicable. This is a systematic review and meta-analysis, no ethical issue was involved, thus the ethical approval is not available.

## Consent

Not applicable. This is a systematic review and meta-analysis, participants included in this research were not enrolled by ourselves, relevant data was harvested from other published articles, thus their informed consents are not available.

## Sources of funding

This research is supported by the Young and Middle-aged Doctor Research Project of the Beijing Bethune Public Welfare Foundation (No.Z04JKM2022E036), the China Postdoctoral Science Foundation (GZC2023156) and the Natural Science Foundation of Hunan Province of China (2024JJ6664).

## Author contribution

M.C.: data curation, formal analysis, investigation, software, visualization, writing – original draft, and writing – review and editing; T.Y.: data curation, formal analysis, investigation, and methodology; X.M.: data curation, formal analysis, investigation, and methodology; Z.W.: writing – original draft; W.W.: conceptualization, funding acquisition, methodology, project administration, resources, supervision, validation, and writing – review and editing.

## Conflicts of interest disclosure

The authors declare that the research was conducted without any potential conflict of interests.

## Research registration unique identifying number (UIN)

Research Registry: reviewregistry1821

PROSPERO: CRD42024501306.

## Guarantor

Wenlong Wang.

## Data availability statement

The data of this meta-analysis and systematic review is available online. Further inquiries can be directed to the corresponding author.

## Provenance and peer review

Not commissioned, externally peer-reviewed.

## Supplementary Material
