# Supplementary figures and images for: The preferred surgical choice for intermediate-risk papillary thyroid cancer: total thyroidectomy or lobectomy? A systematic review and meta-analysis
Source: Int J Surg. 2024 May 13;110(8):5087–100. doi: 10.1097/JS9.0000000000001556 (PMC11325972; doi:10.1097/JS9.0000000000001556)

## Identification of studies via databases and registers

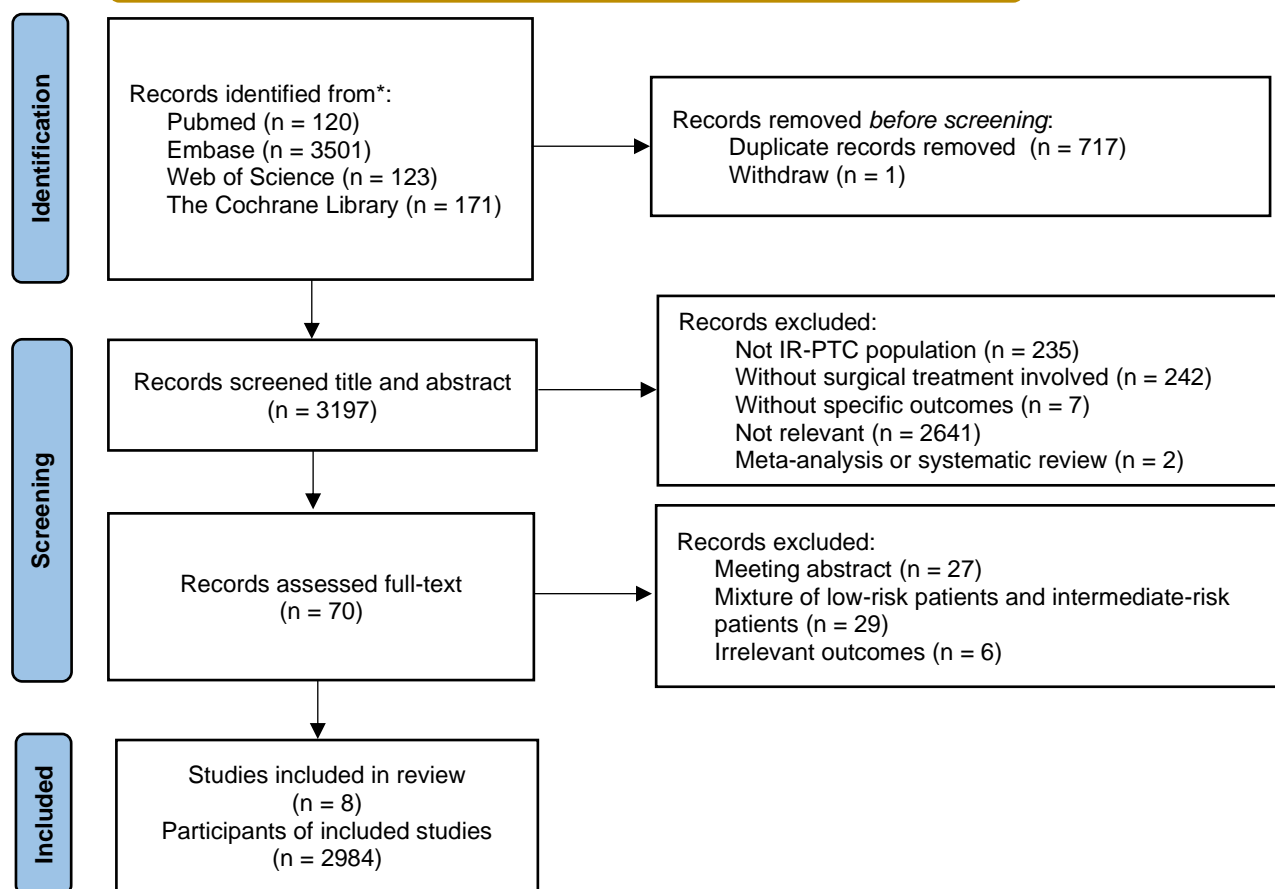

Supplement: Supplementary file 2 [file js9-110-5087-s002.pdf]
